# Collaboration in neuroscience: the young PI perspective

**DOI:** 10.1111/ejn.13226

**Published:** 2016-03-31

**Authors:** D. Belin, A. Rolls

**Affiliations:** ^1^ FENS‐Kavli Network of ExcellenceEurope‐wide; ^2^ Department of PharmacologyUniversity of CambridgeCambridgeUK; ^3^ Technion‐ Israel Institute of TechnologyHaifaIsrael

The 21st century was deemed to be the century of the nervous system and associated diseases. We entered the century with the grand ambition to gain a richer understanding of ourselves by uncovering the mysteries of the human mind and develop new ways to prevent and cure brain disorders. However, these aims remain, as yet, unfulfilled and disorders like Alzheimer's, schizophrenia, autism, depression, addiction and epilepsy still represent major health, economic and social burdens.

The challenging complexity of the nervous system has necessitated the partitioning of the field into very specialized sub‐disciplines; any given laboratory aims to understand a specific layer of information processing in the brain: from molecules to behavior, from networks to computation, from cells to cognition. This “fragmented” approach to the brain has been driven by (i) the necessity to uncover and accumulate basic knowledge about the various levels of integration of the brain and (ii) the overwhelming complexity that precludes any single researcher from approaching the whole problem from top to bottom. However, in the last decade we might have reached a knowledge threshold, beyond which these “distinct” fields of neuroscience ought to be merged to understand how molecular mechanisms in neural networks orchestrate sophisticated, adaptive behaviors and cognitive processes, and how they go awry in neuropsychiatric disorders.

This is where our individual limitations impinge on us and multidisciplinary approaches become necessary. Few laboratories can, on their own, begin to approach these questions, which demand a multi‐systems, multi‐disciplinary approach by their very definition. No single PI will be, simultaneously, an expert in computational neuroscience, experimental psychology, fMRI, patch‐clamp and RNAseq, which are only a small subset of the tools required to take such a comprehensive view. Science should be driven by hypotheses, which should not be limited to the techniques present in the lab. To solve these big questions, and often to obtain the funding for these endeavors, we must work together. Collaboration offers the unique opportunity to expand the knowledge base of the members of your laboratory, train people in new techniques and open new ways of thinking. Moreover, collaboration is also an excellent strategy to disseminate your knowledge, as co‐authored papers tend to be cited more frequently (Adams, 2012).

As members of the FENS‐KAVLI network of excellence, representing neuroscientists at the early and mid stages of their career, we feel that we are a generation that is used to collaborations and greatly appreciates their importance. Many of us were educated in a generation that witnessed large collaborative projects, such as the genome project, that changed the mindset of scientists and the scientific culture. We often work in open spaces designed to foster collaboration, belong to multidisciplinary networks or part of integrative research centers. We have been witnessing this change to collaboration across most scientific disciplines as more scientists are working and publishing together. An issue of *Nature* today has a similar number of Letters to an issue published 60 years ago, but at least four times more authors (Greene, 2007; Adams, 2012).

As a young PI, you are about to, or will eventually, engage in collaborative research projects from which you will gain a lot of experience, expertise and generate scientific output you would not have been able to achieve on your own. In some cases, you will initiate the collaboration and, in others, the collaboration will find you. Sometimes you will contribute to the concept and, other times, you will provide a unique expertise and technique that a collaborative research project would rely on. Each case is different and most importantly, it is a human adventure, involving not only yourself and your collaborator, but members of each lab and some of the joint resources. It is therefore very important to be well equipped to tailor your collaborative projects to fit your needs and your working habits.

Eventually, some collaborations can turn into a lifelong journey, others may be a short fling and hopefully, only a few of them will become a source of mutual disappointment, especially provided you can equip yourself either to avoid them or manage them better. In this opinion piece, we put forward points for consideration and a road map to visualize the process of establishing a collaboration as a young PI. We suggest a decision tree (Fig.**** 1) based on simple questions you should ask yourself and your collaborator so that you can make informed decisions about the collaboration you are considering. We also discuss some basic management “rules” for the collaboration that may help you avoid potential traps. Indeed, collaborations can pose challenges to young PIs that are very different from those of established labs.

**Figure 1:**
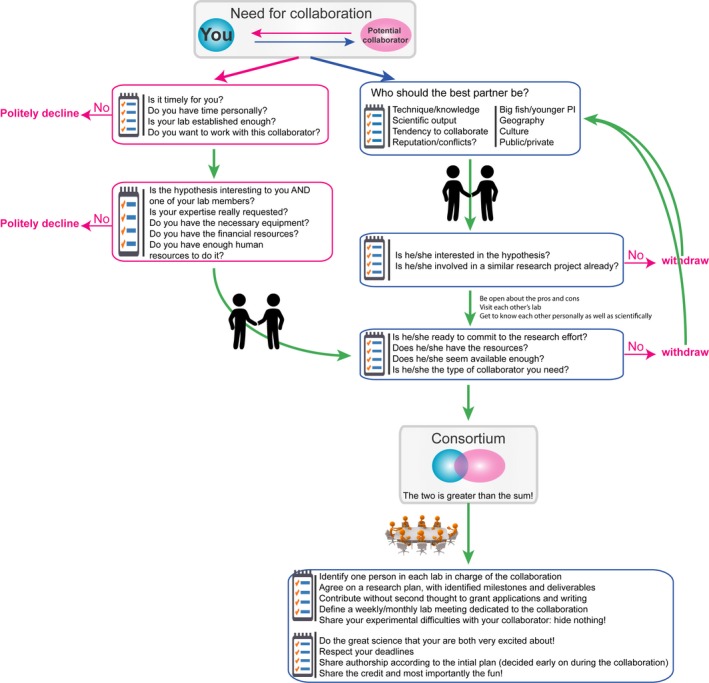
Decision tree of a collaboration process. The tree is divided into two general scenarios as described in the text. On the left, is a suggestion for making appropriate and decisions when you are approached for collaboration. On the right, is a suggestion for your process when looking for a collaborator. Finally, at the bottom part of the figure is a set of suggestions for establishing and coordinating the collaboration. Each question/point is also discussed in the main text. DB has successfully published more than 10 papers stemming from collaborative work and raised more than €1M through collaborative projects (including from the French Agence Nationale de la Recherche, the France Parkinson foundation). AR is a strong believer in collaborations and published 11 joint papers, seven of them with the same collaborator. She is currently involved in two funded collaborative projects. DB and AR are now collaborating on a multidisciplinary, international project.

## On being approached for a collaboration

Some people, especially at the early stages of their career, feel pressure to agree to collaborate in order to avoid uncomfortable confrontation and end up finding themselves in an even more complicated situation when they reach a milestone and can't deliver. This may be even worse if you are approached by a senior researcher. In this case, you may feel that your career prospects depend on their opinion and you may believe that refusing such collaboration is not really an option. However, you should keep in mind that committing to collaborate when you are not ready will ultimately lead to a failure to deliver on time and will reflect negatively on you and your lab. Thus, the first real decision to make when approached for collaboration is whether you and your lab are ready. Often enough, an opportunity to collaborate will occur very early in the career of a young PI, even before they have fully established their lab, instilled a working and intellectual culture which they want to be the “trademark” of their lab and had a chance to appreciate the strengths and limitations of their management capabilities, technical skills and human resources. While an early collaboration could be a great opportunity to secure more funding or boost the productivity of the lab, it may be dangerous to be too hasty and commit to a collaboration without a good estimate of whether you can actually deal with what it entails. Additionally, starting a collaboration too early may also impinge on the culture and working habits you want to establish in your lab. Labs have different cultures and if you have not given your lab enough time to establish its own identity, the culture of the other, more established, lab may leave a permanent imprint on yours. Therefore, timeliness is a major component of choosing whether to accept a collaboration or not. You need to be honest with yourself about your ability to deliver and your desire to collaborate. If the timing is not right, you can explain this to your colleague. If it is the right time, there are many criteria on which to base your decision to accept or decline to collaborate. Below are some examples of questions you should ask yourself to help you decide:

- Are you interested in the hypothesis? If you are not excited about the hypothesis, you will not invest enough time and energy for the collaboration to succeed. Be honest with yourself; you are a human being and if the intellectual incentive is not there to motivate you, you will not work for it to the degree your collaborator would expect you to. You owe them to share their interest and commit proportionally.
- Does the hypothesis appeal to at least one other member of your lab and does this member have enough time to dedicate to the joint research project? You will not be alone in this collaboration. It is important you share the prospect with your lab members and gauge who would be happy to share some of their equipment for, or dedicate some of their time to, the collaborative project.
- Does the hypothesis fit with your overall work plan? Obviously, as a young PI, you must be careful not to diversify your research too much too early so that you can establish your name in your field first and only then expand your research interests.
- What will the collaboration contribute to you? You have to adopt a pragmatic approach with regards to collaboration opportunities, and always consider whether the outcome of this collaborative work will be worth the investment as compared to a similar investment towards your in‐house projects. In case it is a very well established, recognized colleague who approaches you, you also should consider whether the collaboration is set up such that you and your lab will receive credit for the results. This is a delicate thing to consider, but confronting this issue early will help you avoid headaches further down the road.
- How many resources will it take from you? Time, money, work hours? As a follow‐up, or prerequisite to the previous point, it is important to actually quantify the “cost” (or “opportunity cost”) of a collaboration. For instance, the resources that will be used towards the collaborative project will not be used for your own projects.
- Do you have the required expertise? You should make sure that you can carry out the experiments pertaining to your side of the project without installing new equipment, going though major trouble‐shooting or establishing a brand new technique. As a young PI, you may not be able to invest that much time and effort into something that is not your primary area of expertise.
- Do you want to work with this person? The hypothesis may be appealing, the timing is right and you are confident that you have the expertise to deliver. However, there is a “last but not least” consideration concerning your desire to work with this person. Many factors could contribute to this decision, but you may simply have a “gut feeling” that you don't want to work with this potential collaborator. Listen to your instinct! You will eventually both be better off.
- How did his/her previous collaborations work out? In case you are ready to collaborate with this person, it is important to feel comfortable in trusting a new close collaborator and you may want to enquire whether his/her previous collaborations worked out. With collaborative projects come joint‐authorship, and PubMed can tell you a lot about their way of handling collaborations. This will also help you to approach the fundamental question of how to share the credit and the associated authorship issues (see section about setting‐up the collaboration) were you to initiate the collaboration.

In conclusion, when you are asked to collaborate, don't assume that you must agree. Taking on obligations that you cannot deliver may be much worse than refusing the project in the first place. Consider the impact of the collaboration on your time, resources and overall goals of your laboratory. If you conclude that collaboration is the right way to continue, you may find the “Guidelines to set up a successful and healthy collaboration” useful.

## If you are the one seeking to collaborate

When you are the one initiating the collaboration, you have more freedom to choose your partner but it also places a greater responsibility on you. You need to identify the right partner, convince them to join the project and then, navigate the collaboration for successful and effective interaction. Here are a few points to consider when selecting your partner.

- What do you need from your collaborator: It is important to define what are you looking for in your collaboration. Is it an opinion, an experimental expertise, access to a database?
- Are you looking for an academic collaborator or an industry partner? If you are seeking an industry partner, it is a good idea to get the advice of your institute's business development center. A partnership with industry is often a great opportunity to translate your ideas into practice and may open many funding opportunities. However, industry has different rules, both in common scientific practice and their motivation to publish the data.
- Working with a friend or a stranger? A study that measured the proportion of authors who had worked with each other previously found that if the PIs had a previous history of collaboration, their project was much more likely to be successful than if they had never written a paper together before (Cummings & Kiesler, 2008). On the other hand, papers in high‐impact journals have a strikingly lower proportion of these repeated interactions than do papers in lower‐impact journals (Guimera *et al*., 2005).
- Does the collaboration require geographic proximity? Intuitively, one may say that proximity is a great advantage, as it will allow more immediate and direct communication. However, a study that analyzed a sample of 4.2 million papers published between 1975 and 2005 divided universities into tiers based on the number of citations their researchers achieved and found that teaming up with someone from another institution of the same or higher tier reliably produced more highly cited publications (Whitfield, 2008).
- Do you prefer to work with a well‐known scientist (vertical collaboration) or with someone around your academic stage (horizontal collaboration)? Obviously, working with a well‐known scientist comes with many advantages. It is more likely that things will work, that the project is funded and that the publication is accepted in a higher tier journal. One major risk of such vertical collaboration is the likelihood that, no matter how much you put into the collaboration and how reliable and supportive your collaborator will be, the findings may become associated more with this well‐known scientist than with you. This may not be the case if you both come from very distinct fields, so that your contribution will be evident in your scientific community. A special case of such vertical strategy is collaborating with your former PI or your mentor (see later).

Once you have considered all the alternatives and identified a potential collaborator, you will need to ensure that they are the right fit because establishing a collaboration with someone who will not be dedicated to the project can be very frustrating. Here are some points to explore when you communicate with the potential collaborator:

- Does he/she have an interest in the hypothesis? If you think that the hypothesis does not elicit excitement from your potential collaborator, it may not be a good idea to “talk them into it”. Eventually, they will likely end up finding excuses for inaction. However, you should also remain aware that you may not always be able to reliably estimate others' enthusiasm; each person has their own communication style and personality. This may be especially relevant in cross‐cultural collaborations.
- Is he/she involved in a similar project? Although it may seem obvious, it is actually a tricky point to consider. It is obviously a matter of ethics and general courtesy, but it is not unheard of that converging ideas emerge at the same time, and involve the same labs. You may want to ensure that the specific scientific question you want to address with them does not compete, or interfere with, other projects in their own lab or other labs with which they collaborate.
- Does he/she have the time and resources to commit? Some people will tell you directly what they can do, while others may over‐estimate their ability or under‐estimate their commitments. Thus, it may be helpful to generally break down the stages of the project to ensure that your collaborator fully acknowledges the extent of the commitment.
- Do you communicate/interact well with the person? If you have the option to meet in person and spend some time discussing the details of the project, you may get a better idea of how well you communicate and interact. Conferences can be a great opportunity to meet your potential international collaborator.

If, addressing these questions, you conclude that you are ready for this collaboration, then it is now time to think about the structure and organization of the effort. Below are some guidelines to help you set up a successful collaboration.

## Guidelines to set up a successful and healthy collaboration

A successful collaboration requires transparency, honesty and commitment. In the process of setting up the collaboration, you have already made sure that this colleague is the right collaborator for the project and that you get along well enough to commit to each other for the greater good: better science than either of you could do on your own. Now it is time to implement a formal strategy that will consolidate the good will of each of the individuals involved and ensure a healthy and successful collaboration. This should start with a first formal meeting. This can be either a meeting between the PIs or a larger meeting, gathering as many members as possible from each lab who are likely to be involved in the project. If possible, it is better that you all meet in person for the first meeting because it will provide the foundations for a close relationship between the different members of each lab. For follow‐up meetings, you can rely on video‐conferencing, which has changed the way we can interact on a regular basis, saving both time and money. Of course, you can't have that fancy drink in a pub that follows a meeting and fortifies the relationship, but there will be opportunities for it. During this first meeting, you may want to formally discuss each of the following aspects of the collaboration:

- Identify the lead person in each lab, who will be in charge of the project. Of course, the PIs will be in charge of the overall supervision of the project, but it may be a good idea to delegate the ongoing management of the project to one member in each laboratory who can make sure it runs smoothly.
- Agree on a research plan and identify precise objectives, milestones and deliverables for both sides. This will be the core of the meeting and can also serve as the basis of a joint grant application. This discussion will help you to plan carefully and think through the commitments made by each contributor. It will help to identify potential obstacles or caveats in the structure of the partnership that need to be addressed in the earliest stages of the collaboration.
- Start with a small, pilot study. This will allow you to test the water: see how well you work together, adjust the ground rules and the communication strategies. If this does not work, you can withdraw before getting too invested.
- Determine the criteria to be used to assign authorship. This is very important. This discussion should not only cover how the PIs will share their senior authorship but also the criteria that will be used for dividing authorship. There are international guidelines regarding authorship that should be respected to avoid any difficult conflict when you are ready to publish your results. This discussion should be extended to intellectual property, which is more often dealt with by institutions than individual labs.
- Plan regular meetings to follow up on the collaboration. Meet even if you don't have important updates. This keeps the project going during “down” times and prevents you from losing track of the process when data accumulates.
- Establish the work format. How do you communicate (e.g. do you copy everyone on each email)? How do you handle data sharing (mutual data storage)? This is an important point to discuss, especially at a time when more funding bodies require that data be shared. There are many communication tools and several project management tools online that allow everyone to follow the progress of the collaborative project. It may be useful to setup “alarms” in your calendar based on the estimated progress of the project.
- Determine how to handle problems when they arise. Communication is key. If you notify your collaborators about potential delays and problems along the way, you allow them to prepare ahead, so even if it causes some inconvenience, it allows your collaborators to continue to rely on you.
- Discuss funding alternatives and load distribution. Collaborations are opportunities for funding. However, you need to plan the budget even more carefully than you would with your own grants because it will be much harder to change things once the money is divided.
- Provide an agenda prior to meetings (even if held on Skype) and take a few minutes following the meeting to keep track of strategic decisions jointly made and the progress of the collaboration. This will not only help you track and drive the project, but also save you time in case you have to provide progress reports to a funding body.

As in every relationship, communication is a key for collaboration. Once you start working together, keep in mind that you need to be responsible for your deadline. Although anyone may fall behind from time to time, it is crucial to inform your collaborators when you do! It is also a good idea to share a periodic update even if things are going well (add a reminder to your calendar). Often a short email will alleviate uncertainty on the other side.

## Declining a collaboration

It may feel uncomfortable to decline an invitation, especially one that reflects a desire to build a relationship with you or a need for your skills/expertise. However, it is even worse to get involved in a collaboration that you will keep trying to get away from or be miserably reminded of your bad decision. Explain your situation; try to be as honest and forthcoming about your reasons as possible without hurting anyone's feelings. If your potential collaborator cannot see your position, you are clearly better off declining, as their reaction is evidence of a “problematic” personality. In any case, you could offer to help instead of agreeing to collaborate.

## Networks and large group collaborations

Most of this piece refers to small group collaborations. Large collaborations and networks have different rules and are often characterized by a dynamic set up of collaborators that can change along the way. In some cases, joining an established network will take some of the pressure off of you because there are many people involved and tasks are often more distributed. Also, such networks tend to have a coordinator who can buffer many of the personal complexities that may arise when you are working with a small group of people. Being the one in charge of such a network can offer a young researcher some advantages and may be a great networking opportunity, but it can be extremely demanding and put you in confrontation with others far too soon.

## Collaborating with your past mentors

There is a general pressure from the established system to demonstrate, as a young PI, that you are independent, capable of publishing without your PhD or post‐doc supervisor. It may also reflect your own need to develop as an independent researcher, however, if you get along well with your previous mentor and have the opportunity to continue to collaborate with them, it may just be the right thing for you. As with every other collaboration, just be honest with yourself and communicate. One of the first commonalities we found as members of the FENS‐Kavli Network of Excellence is that we all had the chance, at some point in our careers, to meet a great supervisor/mentor.

## Conclusion

Overall, there are many types of collaboration. Some begin with people, others begin with an idea. Sometimes a great personal interaction motivates a group of scientists to start working together, searching for the right project. In other cases, the idea is the motivation to start looking for the right partners to collaborate with. Either way, when you know you are ready for it, embarking your lab in a collaborative project is a unique human adventure from which you will gain expertise and knowledge beyond the technical expertise brought about by the collaborating laboratory. It will, in some cases, be the starting point of a life‐long collaboration that will contribute to your science and shared success. It depends on you to find the right partner and to be the right partner to make this unique scientific and inter‐personal experience a success!

## **Acknowledgements**

We would like to thank Nathaniel L Green for his comments and edits. DB is supported by the Wellcome Trust and the University of Cambridge. AR is supported by CIG and Adelis Foundation. DB has successfully published more than 10 papers stemming from collaborative work and raised more than €1M through collaborative projects (including from the French Agence Nationale de la Recherche, the France Parkinson foundation). AR is a strong believer in collaborations and published 11 joint papers, seven of them with the same collaborator. She is currently involved in two funded collaborative projects. Both DB and AR are now collaborating on a multidisciplinary, international project.
